# CartograPlant: bridging genomic, phenotypic, and environmental data to advance plant resilience and eco-evolutionary insight

**DOI:** 10.1093/genetics/iyag060

**Published:** 2026-03-06

**Authors:** Brandon M Lind, Irene Cobo-Simón, Meghan Myles, Gabe Barrett, Emily Grau, Risharde Ramnath, Vlad Savitsky, Jill L Wegrzyn

**Affiliations:** Department of Ecology and Evolutionary Biology, University of Connecticut, Storrs, CT 06269, United States; Institute for Systems Genomics, University of Connecticut, Storrs, CT 06269, United States; Department of Ecology and Evolutionary Biology, University of Connecticut, Storrs, CT 06269, United States; Departamento de Ecología y Genética Forestal, Instituto de Ciencias Forestales (ICIFOR), Instituto Nacional de Investigación y Tecnología Agraria y Alimentaria—Consejo Superior de Investigaciones Científicas (ICIFOR-INIA, CSIC), Madrid 28040, Spain; Department of Ecology and Evolutionary Biology, University of Connecticut, Storrs, CT 06269, United States; Department of Ecology and Evolutionary Biology, University of Connecticut, Storrs, CT 06269, United States; Department of Ecology and Evolutionary Biology, University of Connecticut, Storrs, CT 06269, United States; Department of Ecology and Evolutionary Biology, University of Connecticut, Storrs, CT 06269, United States; Department of Ecology and Evolutionary Biology, University of Connecticut, Storrs, CT 06269, United States; Department of Ecology and Evolutionary Biology, University of Connecticut, Storrs, CT 06269, United States; Institute for Systems Genomics, University of Connecticut, Storrs, CT 06269, United States

**Keywords:** data integration, data interoperability, meta-analysis, biodiversity informatics, plant adaptation, GWAS, GEA, FAIR

## Abstract

Climate change is threatening plant health and productivity at all spatial scales, and these impacts are further compounded by the rising incidence of invasive pests and pathogens. Effectively addressing these challenges requires a comprehensive understanding of plant demography as well as the mechanisms and drivers of adaptation. Achieving this understanding requires the integration of physiological, ecological, and genetic datasets. However, such integration is often hindered by disconnected data sources, inconsistent metadata standards, and limited tools to link, analyze, and visualize multi-dimensional datasets in a unified framework. Addressing these hurdles is critical to advancing the understanding of species responses to environmental change and developing informed strategies for conservation, restoration, and adaptive management. CartograPlant (https://cartograplant.org) is a web-based interactive application which facilitates the visualization and analysis of genotypic, phenotypic, and environmental data, as well as associated metadata, from georeferenced individuals. Developed as a Tripal module, CartograPlant addresses a critical gap in biological data integration by enabling users to explore complex eco-evolutionary patterns across space and time. Recent updates have expanded its data sources, improved interoperability, and introduced NextFlow pipelines alongside new tools for the integration and analysis of these data. CartograPlant offers a scaleable, flexible, and continually updated platform for researchers, conservationists, land managers, and plant breeders to better understand and mitigate the impacts of global change on plant biodiversity, accelerate resilience in breeding programs, and inform data-driven decisions in agriculture and ecosystem management.

## Introduction

Globally, plant species face pressures from habitat loss, native and invasive pests and pathogens, and climate change (Urban 2015, 2024; Nadeau et al. 2017). The upcoming century is forecasted to bring increasingly novel climates with no historical analog (Mahony et al. 2018; Smith et al. 2022; Ordonez et al. 2024; Kerr et al. 2025), which is expected to disrupt relationships between locally adaptive genetic variation and environmental optima of many species (Aitken et al. 2008; Wilczek et al. 2014; McGraw et al. 2015; Browne et al. 2019; Fréjaville et al. 2020; Anderson et al. 2025). Such mismatches exacerbate environmental stresses on plants, resulting in increased susceptibility to both native and invasive pests and pathogens (Hellmann et al. 2008; Mainka and Howard 2010; Velásquez et al. 2018; Hartmann et al. 2022) as well as reductions in species' genetic diversity (Exposito-Alonso et al. 2022; Shaw et al. 2025).

Addressing these challenges requires a comprehensive understanding of plant ecology and evolution, as well as the ability to predict responses to global change (Ehrlén and Morris 2015; Díaz et al. 2019; Johnston et al. 2019; Capblancq et al. 2020; Lee-Yaw et al. 2022; Anderson et al. 2025). Recent advancements in genomic sequencing and high-throughput phenotyping offer excellent opportunities to address these knowledge gaps (Mir et al. 2019; León et al. 2023; Bernatchez et al. 2024; Nguyen et al. 2025). Knowledge of neutral population structure as well as the genetic variation that confers resilience to climate change, pests, and pathogens can be leveraged for tailored management actions to enhance health and productivity at both the species and ecosystem level (Fraser and Bernatchez 2001; Funk et al. 2012; Barbosa et al. 2018; Thorogood et al. 2023). However, to translate these insights into effective management, it is necessary to integrate diverse data types and approaches to capture the complexity of plant responses to environmental change. Such integration can also enhance our understanding of plant adaptations (Sork et al. 2013; Rellstab et al. 2015; Josephs et al. 2017; Carvalho et al. 2021; Volk et al. 2021; Lasky et al. 2023).

Three main data types are used to understand plant adaptations to environmental variation (Sork et al. 2013): genomic data (i.e., DNA nucleotide and structural polymorphism data as well as the intermediate, reference, and pangenome assemblies to which they are mapped), geospatial data (i.e., georeferenced climatic and environmental data), and phenotypic data, which includes morphological, developmental (e.g., phenology and ontogenic traits), physiological, molecular (e.g., transcriptomic, proteomic, metabolomic, and epigenetic traits), and categorical traits (e.g., infection status, survival, and sex). Although individual genomic and phenotypic datasets have grown in prevalence and volume, there is increasing recognition of the need for well-curated spatiotemporal metadata to enable future data reuse (Toczydlowski et al. 2021; Crandall et al. 2023; Leigh et al. 2024; Forsdick et al. 2025). Well-curated datasets enable a deeper understanding of adaptive and demographic dynamics within species by facilitating reanalysis, meta-analysis, and the analysis of combined datasets. Indeed, integrative analyses highlight the value of data reuse as a powerful framework for uncovering the eco-evolutionary processes that shape genetic diversity across space and time (Leigh et al. 2021; Hoban et al. 2022; Schmidt et al. 2023).

Despite the benefits offered by biological data integration, its implementation can be challenging. Storing and integrating data from diverse sources (genotypic, phenotypic, and environmental) can be difficult due to inconsistent reporting standards and decentralized repositories (Deng et al. 2023). The FAIR (Findable, Accessible, Interoperable, and Reusable) principles provide guidelines to address these issues, promoting machine-actionable data through readable metadata, persistent identifiers, standardized data formats, and the enforcement of community-curated ontologies (Wilkinson et al. 2016). However, FAIR implementation for non-model organisms faces additional obstacles due to a combination of biological, technical, and infrastructural challenges. For instance, many non-model organisms lack reference genomes, or consistent versioning where one exists, and rely on intermediate assemblies that are seldom archived (e.g., RADseq contigs or *de novo* assembled transcriptomes). These limitations are compounded in multi-omics contexts, where differences in methods, technology, and data types complicate data integration and machine-actionable use (Jamil et al. 2020; Hao et al. 2025). While ontology-driven frameworks like MIAPPE (Minimum Information About a Plant Phenotyping Experiment) have indeed improved standardization for trait data, their adoption has not been uniform (Papoutsoglou et al. 2023). Despite these efforts, the widespread adoption of FAIR principles has remained limited, especially for data associated with non-model organisms.

While existing repositories for data archival provide valuable access to plant research datasets, they remain fragmented and distributed across several specialized databases and repositories. For example, NCBI and Ensembl Plants (https://plants.ensembl.org/index.html) provide access to reference genomes and annotations for many taxa. Genomic variant data are hosted at the European Variation Archive (https://www.ebi.ac.uk/eva/). Dryad provides long-term archival of genotypic and phenotypic datasets, while resources such as the Global Biodiversity Information Facility (gbif.org) provide global species-occurrence data. Web-based tools such as GWAPP (Seren et al. 2012) and easyGWAS (Grimm et al. 2017) offer integrated approaches to genotypic and phenotypic data but are primarily designed for use with a few model species and do not support the integration of multidimensional, georeferenced datasets that include environmental variables. As a result, unified platforms for the integration, visualization, and analysis of large heterogeneous datasets remain limited.

**Fig. 1.**
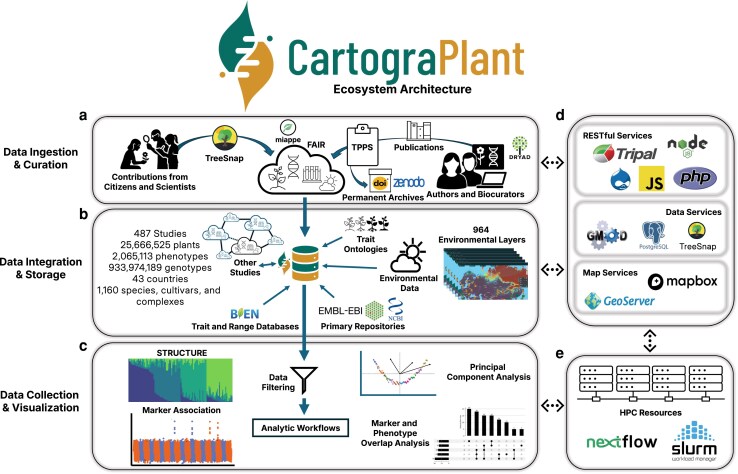
The CartograPlant ecosystem architecture facilitates eco-evolutionary insight. CartograPlant is web-based interactive application composed of interconnected modules that support integrative, reproducible analyses though scalable, standards-based infrastructure and services: a) *Data Ingestion & Curation*: Phenotypic, genetic, and environmental data, and the associated metadata and experimental design information, are collected from both citizen science platforms such as TreeSnap as well as trained biocurators through the TPPS pipeline (Tripal Plant Population Submit, see Fig. 2). These data are curated using community standards such as the MIAPPE (Minimum Information About A Plant Phenotyping Experiment) and FAIR principles (Findable, Accessible, Interoperable, and Reusable). When data has not been previously archived, CartograPlant offers the option to create a Zenodo archive. b) *Data Integration & Storage*: Ingested data are incorporated into a Chado database schema that integrates current study information and metadata with other ingested studies, databases, repositories, ontologies, and environmental layers (see Table 1). c) *Data Collection & Visualization*: Registered users of CartograPlant can create saved workspaces that retain raw data linked to a collection of individuals as well as the output files and figures generated through subsequent analysis. d–e) *Services*: CartograPlant's ecosystem architecture is enabled by RESTful, data, and map services alongside high-performance computing (HPC) resources and NextFlow pipelines to support reproducible and scalable data processing. CartograPlant is available online at https://cartograplant.org.

To address these gaps, CartograPlant (https://cartograplant.org) was developed as a Tripal module (Falk et al. 2018) to facilitate the integration, visualization, analysis, and meta-analysis of genotypic, phenotypic, and environmental data for georeferenced plants. The Tripal toolkit, which supports numerous databases worldwide, provides a common framework for the storage of genomic, genetic, and breeding data, reducing duplication of effort and improving interoperability (Sanderson et al. 2013; Staton et al. 2021). CartograPlant (Fig. 1a–d) extends the original CartograTree framework (Vasquez-Gross et al. 2013; Herndon et al. 2016)—which was developed for visualization and and analysis of biological and environmental data from georeferenced tree species—and broadens its capabilities to support a wider diversity of plant species. Specifically, the platform has improved its interactive web-based geospatial interface and has continually added analytic tools and NextFlow pipelines that leverage High-Performance Computing (HPC) resources for real-time analysis. While such workflows typically require command-line proficiency and direct interaction with HPC resources, CartograPlant's interface automates these steps, allowing users to launch and monitor analyses entirely through the web platform. Here, we review the functionality of CartograPlant, including data ingestion and integration methodologies, data availability, and supported analyses.

## Data ingestion and integration

CartograPlant is designed to create FAIR datasets from existing and ongoing studies of plant populations that integrate genotype, phenotype, and environmental data in a geospatial context. Datasets are sourced from studies of plants on the landscape, in common gardens (including reciprocal transplant experiments), and in controlled environments such as greenhouses and growth chambers. CartograPlant is designed to host FAIR datasets arising from diverse areas of plant research including landscape genomics, quantitative genetics, ecophysiology, and conservation biology. Closely associated with the TreeGenes database (https://treegenesdb.org/), CartograPlant has a unique role in providing a platform for the ingestion of studies focused on both traditional model- and non-model plant systems.

**Fig. 2.**
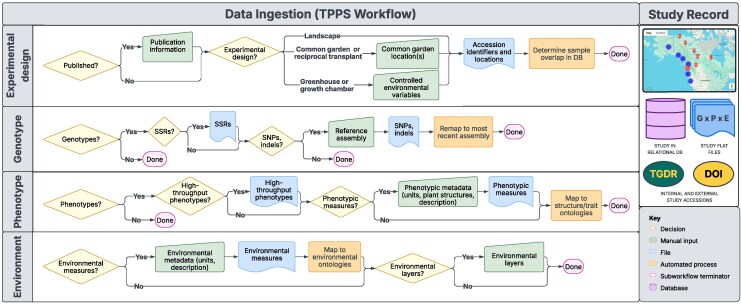
Data ingestion workflows for integrating genotypic, phenotypic, and environmental data through the Tripal Plant Population Submit pipeline (TPPS; http://treegenesdb.org/tpps). This flowchart outlines the structured processes on CartograPlant for ingesting diverse biological datasets, faceted by four primary information sources: Experimental Design, Genotypic Data, Phenotypic Data, and Environmental Data. Each workflow includes decision points (yellow diamonds), manual data input steps (green trapezoids), file upload actions (blue rectangles with curved bottom edge), automated processing steps (orange rectangles), and subworkflow endpoints (purple ovals). This flexible and modular framework ensures consistent and accurate data ingestion and integration across diverse study types. Upon data ingestion, the study record created, is assigned a permanent TreeGenes Database Record (TGDR) number, and can be assigned a DOI. Files are held in association with ontologies within a relational database (DB).

Data ingestion is organized at the level of a study, with a focus on enhancing the utility of shared data objects across studies (Figs. 1a and  2). These objects can include genotyping assays applied across studies, plant populations monitored over time, traits measured at multiple time points or with identical ontological definitions, climatic variables derived from environmental layers, and markers which are reused or re-discovered over time. This effort is supported by a structured import mechanism that adheres to FAIR data standards and combines community-sourced study submissions (including from the authors themselves), published studies imported directly by trained biocurators, and external sources of trait metrics, including mobile applications (e.g., TreeSnap; Crocker et al. 2020) and independent repositories (e.g., anecdata.org; and BIEN, Maitner et al. 2018).

Both direct submissions and internal study biocuration are facilitated by the Tripal Plant Population Submit pipeline (TPPS; http://treegenesdb.org/tpps, Fig. 2). TPPS provides users with a questionnaire that adapts dynamically to user responses and permits the direct upload of genotypic, phenotypic, and environmental data (Table 1). Metadata describing the publication and the experimental design is also collected and is consistent with the MIAPPE *v1.1* standard, which supports the description of studies involving both perennial plants and traditional crop models (Papoutsoglou et al. 2020). Reporting standards in MIAPPE are realized through connections to existing biological ontologies. CartograPlant hosts and actively curates several ontology mappings that describe aspects of genotype, phenotype, and environment (Table 1). Ingested trait and field-measured environmental data include metadata that describes instrumentation, units, scales, timing, and the associated plant anatomical structure(s) (Fig. 1a). This detailed annotation facilitates informed mapping to ontologies. Alternatively, environmental data may be incorporated into a study through the selection of any of CartograPlant's 964 global and regional environmental layers, with metrics from chosen layers systematically associated with the georeferenced coordinates of all studied plants (Fig. 1b). Whether ingested directly or derived from spatial layers, these datasets are standardized for integration. Once a study has been submitted and approved by CartograPlant biocurators, it can be assigned a Digital Object Identifier (DOI) through Zenodo (European Organization for Nuclear Research 2013). If the dataset already has a DOI (e.g., through Dryad) or accession (e.g., through NCBI), CartograPlant will also reference those existing identifiers, ensuring the linkage between the dataset and any associated publication or previously cited data resources. CartograPlant now hosts geospatially contextualized biological and environmental data from more than 400 studies representing over 25.6 million individuals across 1,160 species, cultivars, and hybrid complexes georeferenced in more than 40 countries. These records also span a wide range of eco-evolutionary contexts, and the structured metadata supports integrative cross-study comparisons and synthesis across the breadth of plant science disciplines. A tutorial for the submission of data through TPPS is available online (see Data Availability).

**Table 1.** Data types and subtypes held within CartograPlant as well as the ontologies with which they are described.

Data type	Associated ontologies	Data subtype	Accepted ingestion formats	Retrieval formats
**Genotypic**	Sequence Ontology^a^ (SO), Gene Ontology^b,c^ (GO)	Assembled or unassembled DNA or cDNA sequence	FASTA	FASTA
		Microsatellite	CSV, TSV	CSV
		SNP	VCF, CSV, TSV	VCF
		Indel	VCF, CSV, TSV	VCF
**Phenotypic**	Plant Ontology^d,e^ (PO), Crop Ontology^f^ (CO), Chemical Entities of Biological Interest^g^ (ChEBI), Phenotype And Trait Ontology^h^ (PATO), Plant Trait Ontology^e^ (TO)	Morphological	CSV, TSV	CSV
		Physiological	CSV, TSV	CSV
		Phenological	CSV, TSV	CSV
		Metabolomic	CSV, TSV	CSV
		Expression	CSV, TSV	CSV
**Environmental**	The Environment Ontology^i^ (ENVO), Plant Experimental Conditions Ontology^e^ (PECO)	Point measure	CSV, TSV	CSV
		Layer	Shapefile, geoTIFF, MBTiles	CSV

File extensions accepted for upload of these data are given as well as the extensions with which data can be retrieved.

Abbreviations: CSV = comma-separated values; TSV = Tab-separated values; VCF = variant call format.

^a^ Eilbeck et al. 2005.

^b^ Ashburner et al. 2000.

^c^ Aleksander et al. 2023.

^d^ Cooper et al. 2013.

^e^ Cooper et al. 2023.

^f^ Shrestha et al. 2012.

^g^ Hastings et al. 2016.

^h^ Gkoutos et al. 2018.

^i^ Buttigieg et al. 2016.

Because CartograPlant aggregates studies collected across many years, the imported datasets reflect a wide range of genomic technologies used to identify genetic variants. In addition, advances in genome sequencing and assembly tools now allow researchers to generate increasingly complete and accurate genomic and transcriptomic assemblies. When variants are anchored to a sufficiently accurate and contiguous genomic or transcriptomic reference, it is possible to realign them to new reference assemblies over time. Realigning and storing previously known markers to new assemblies enables the rapid access of marker datasets across all known reference targets, preserves the utility of earlier discovery efforts, and generates consistent identifiers for re-used markers that map onto published names. CartograPlant updates variant datasets by performing automated flank-based remapping of markers to each stored conspecific assembly (implementation described in Supplementary File 1). In addition, while primary sequence repositories like the NCBI's GenBank sequence database (Benson et al. 2018) support the upload of intermediate assemblies including *de novo* transcriptome assemblies, RAD-Seq assemblies, and resequenced amplicons, in practice they are rarely deposited. Recent developments in CartograPlant have emphasized the submission of intermediate assemblies, along with systematic tracking of reference genomes.

CartograPlant's rich metadata availability, enforcement of standards and ontologies, and automated marker remapping facilitate the integration of both summary statistics and raw data. This ensures that data are prepared for downstream analyses, including meta-analysis and the analysis of combined datasets in integration with all (or a subset) of other relevant datasets.

## Data visualization and collection

The CartograPlant documentation (see Data availability) provides video tutorials that demonstrate retrieval, visualization, and analysis of data within the platform. Using real datasets, these tutorials guide users through selection, filtering, analysis, and retrieval of both raw and filtered data.

**Fig. 3.**
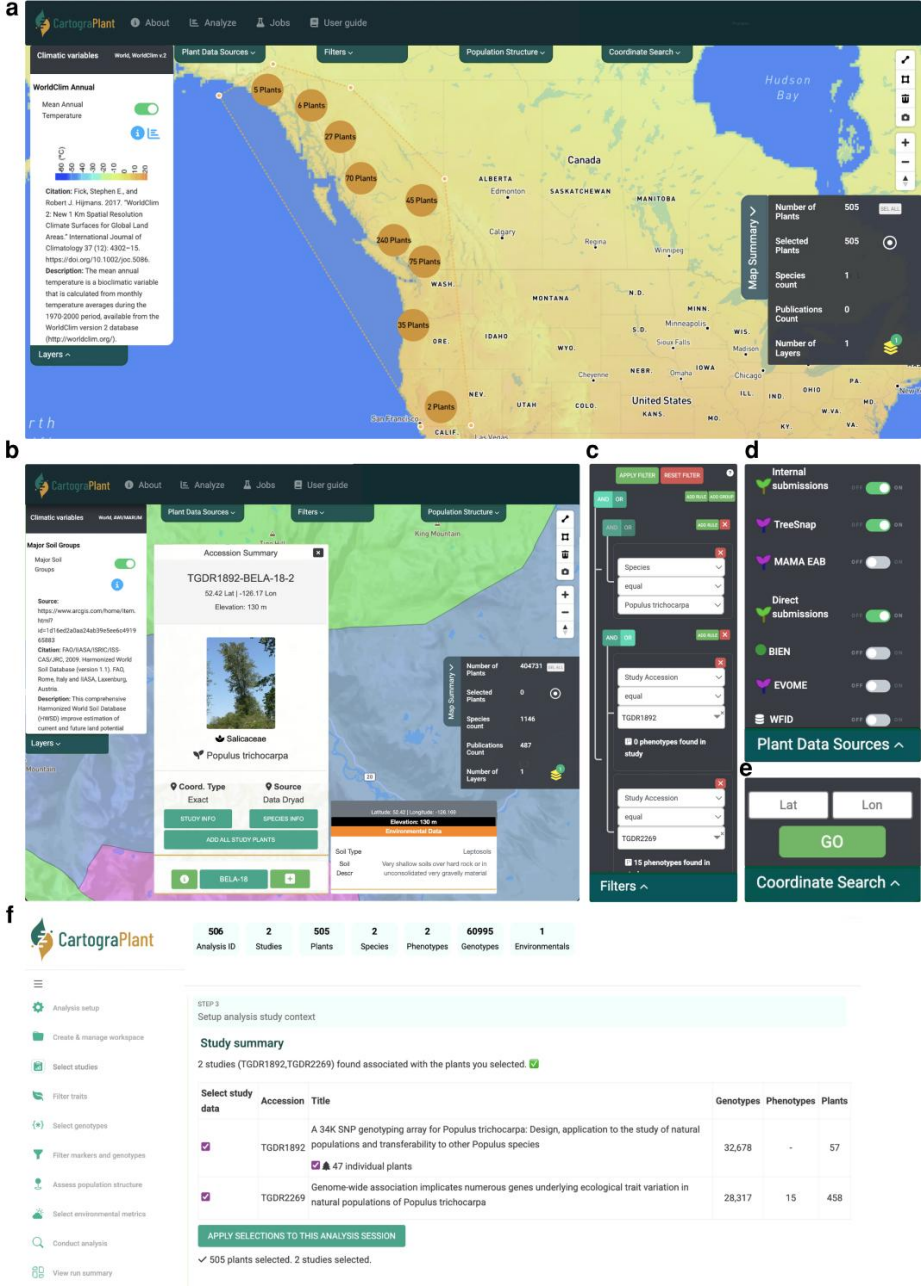
CartograPlant's Browse page enables data filtering and visualization in a number of ways. a) The CartograPlant Browse Page, with the WorldClim2 (Fick and Hijmans 2017) Mean Annual Temperature layer enabled. The Map Summary displays both the number of plants available after filtering and the number of plants collected for analysis. The polygon tool has been used to create a collection (blue bounding polygon). b) The Browse Page with the Harmonized World Soil Database Major Soil Groups layer (Fischer et al. 2008) enabled. A single plant has been selected, enabling the display of that plant's Accession Summary. The Accession Summary displays soil variables from the coordinates of the selected plant, and allows users to add this plant (or all plants associated with this study) to their collection for further use. c) Users can filter displayed data by a number of options with interactive logical operators. Here, filters have been applied such that all displayed plants are members of the species *Populus trichocarpa* and are associated with one of two specific CartograPlant study accessions (TGDR = TreeGenes Database Reference). d) Users can subset displayed data by source. Internal submissions are those made by CartograPlant biocurators, and external submissions are those made directly by authors; TreeSnap = TreeSnap.org. e) Individual coordinates can be searched to zoom to them on the map. f) Collected data described in the Analysis Panel's Select Studies tab. A full tutorial is available online (see Data Availability).

CartograPlant is designed to facilitate data discovery in a geospatial context. All plant accessions are visualized on a world map (Fig. 3a, b). Users can subset displayed data by provenance, study descriptors (title, authorship, accession), taxon (family, genus, species), phenotype (structure, description), and marker type (microsatellites, SNPs, indels) using the Plant Data Source Panel (Fig. 3c) and Filters Panel (Fig. 3d). On the Browse Page, built-in logical operators, available in the Filters Panel, allow complex searches that can be saved to the user's session. Users may also toggle on any of 964 environmental layers, whose values at accession coordinates can be viewed directly. Collections of plants within studies or across studies, can be transferred into the Analysis Panel for refined queries, download, or HPC-supported analysis. The Coordinate Search Panel (Fig. 3e) allows users to move to any location on the map.

When a user selects an individual plant on the Browse Page (map interface), CartograPlant displays an Accession Summary with available data relevant to that individual (see inset of Fig. 3b). These data include the plant's latitude and longitude (and their measurement accuracy), elevation, any included photographs, and metadata about the study or studies with which the plant is associated. In addition, if a user has toggled on any of CartograPlant's environmental layers, the values from that layer at the coordinates of the selected individual are displayed (Fig. 3b). The associated study link available from the Accession Summary displays data from all individuals in the source dataset. When a user selects the Information icon associated with an individual, summaries of measured genotypes and phenotypes at the study level are displayed. This also allows the user to download flat files associated with these data types to facilitate offline analysis.

**Fig. 4.**
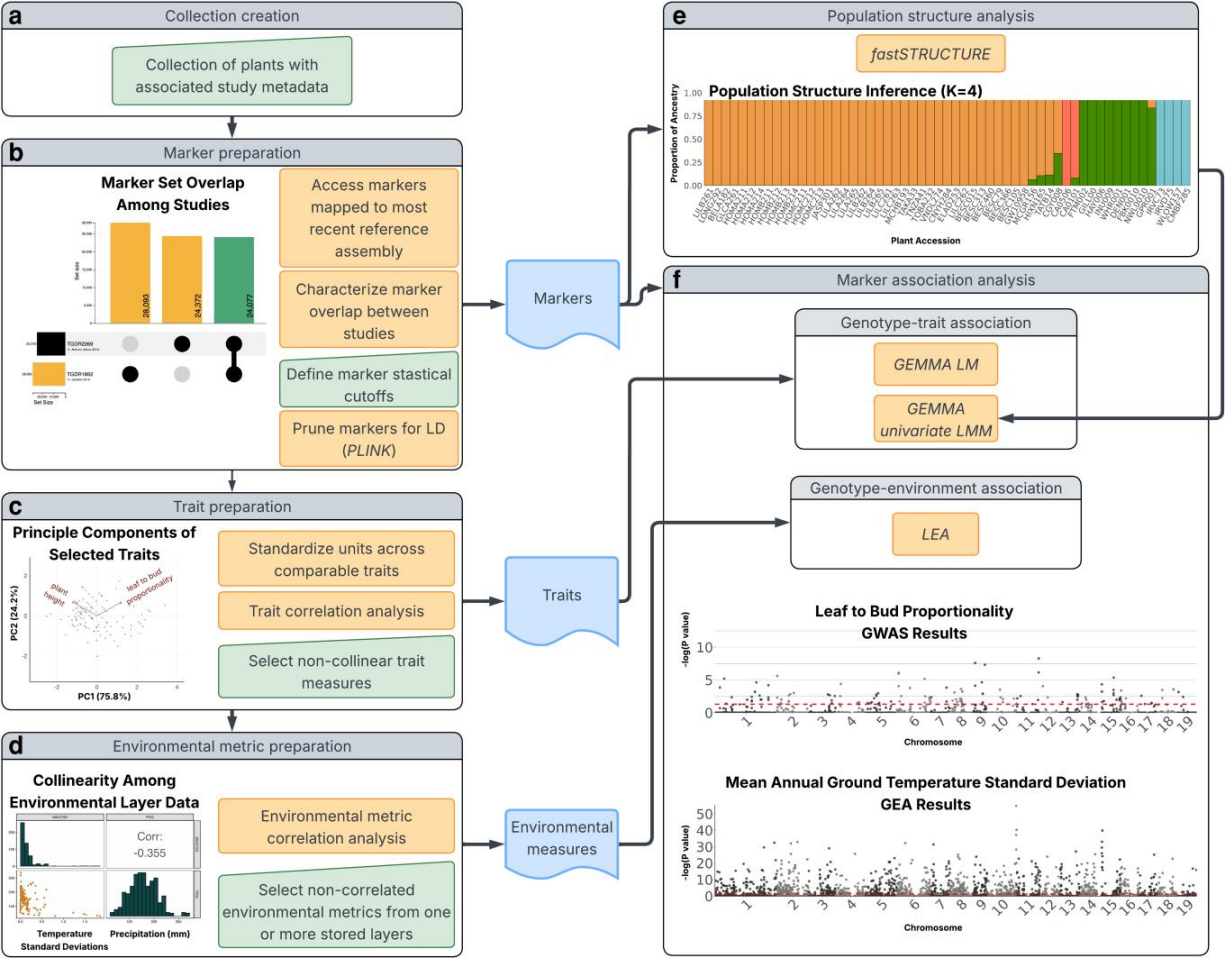
Availability of data filtering, visualization, and analysis methods within CartograPlant's Analysis Panel. a) After creating a collection of plants (see Fig. 3), a user can prepare associated data for analysis. b) Marker overlap between studies is characterized in an UpSet plot. Users can define statistical cutoffs for marker quality and prune their markers for linkage disequilibrium (LD). c) For trait data preparation, users are aided in selecting non-collinear measures with a PCA biplot. Here, the measures *plant height* and *leaf to bud proportionality* have been selected. d) For environmental layer data preparation, users are aided in selecting non-correlated measures with a trellis plot. The upper triangular panels display the pairwise Pearson correlation between environmental variables. Panels on the diagonal display histograms of the distribution of each environmental variable. The lower triangular panels display pairwise scatterplots between each selected variable. MAGTSD = mean annual ground temperature standard deviations; PDQ = precipitation of the driest quarter. Once data selection has been finalized, users can perform analyses of e) population structure, f) GWAS, and GEA. Manhattan plots are displayed to visualize GWAS and GEA results. File outputs at any step can be downloaded from a user's workspace. All data in this figure are associated with CartograPlant accession TGDR 1892 (Geraldes et al. 2013) or with accession TGDR 2269 (McKown et al. 2014c).

Registered users can download or analyze data directly within CartograPlant's Graphic User Interface (GUI), which provides HPC-supported access to several custom bioinformatic workflows (Figs. 3e and  4; Table 2). There are several ways to collect accessions for download or analysis: The Accession Summary display, activated when a user clicks on a plant, can be used to collect an individual or all plants associated with an individual's source study or studies. Alternatively, a user can use the polygon select tool, located in the Map Summary panel, to collect accessions for data download or analysis. After collecting a set of plants, users can view information about the studies with which they are associated including the number of plants, the number of measured genotypes, and the number of measured phenotypes per study (Fig. 3f).

**Table 2.** Workflows available within the CartograPlant Analysis Panel, the general methodology they employ, and the software they utilize.

Workflow	Method	Software
Trait correlation analysis	PCA-based visualization of inferred correlation between selected traits	R *v3.6.0*^a^, ggplot2 *v*3.3.6^b^, ggbiplot *v*0.55^c^
Marker remapping	Flank sequence-based remapping	Python *v*3.6.8^d^, samtools *v*1.22.1^e^, bwa *v*0.7.19 (*mem*)^f^, bedtools *v*2.31.1^g^, pandas *v*2.3.2^h^
Marker overlap analysis	Identification of non-unique marker positions across datasets after remapping to a common reference assembly. Results visualized in an UpSet plot	bcftools *v*1.20^e^, UpSet.js *v*1.11.0^i^
Marker and genotype filtering	Filtering of markers, genotypes, or individuals from a dataset based on user-defined statistical cutoffs. Distributions visualized in histograms	bcftools *v*1.20^e^, D3.js *v*5.16.0^j^
Population structure inference	Inference of population structure from genotype data. Results visualized in an admixture bar plot	PLINK *v*1.90^k,l^, fastStructure *v*1.0^m^, R *v*3.6.0^d^, ggplot2 *v*3.3.6^b^
Environmental data distribution and correlation analysis	Visualization of selected environmental variables, including their distributions, pairwise Pearson correlations, and pairwise scatterplots	R *v*3.6.0^d^, psych *v*2.2.5^n^
Marker association analysis	GWAS or GEA for the inference of associations between genotypes and phenotypic or environmental variables, respectively. *P*-values are visualized within a Manhattan plot	GEMMA *v*0.98.3 (linear model, univariate linear model)^o^, R *v*3.6.0^d^, LEA *v*3.6.0 (*LFMM2*)^p^, ggplot2 *v*3.3.6^b^

^a^ R Core Team. 2025.

^b^ Wickham 2011.

^c^ Vu et al. 2024.

^d^ Python 2023.

^e^ Danecek et al. 2021.

^f^ Li 2013.

^g^ Quinlan et al. 2010.

^h^ Pandas 2019.

^i^ Lex et al. 2014.

^j^ Bostock et al. 2011.

^k^ Purcell et al. 2007.

^l^ Slifer 2018.

^m^ Raj et al. 2014.

^n^ Revelle 2025.

^o^ Zhou and Stephens 2012.

^p^ Caye et al. 2019.

## Data analysis

CartograPlant's Analysis Panel provides an interactive GUI-mediated environment for subsetting, integrating, downloading, and conducting bioinformatic analyses of stored datasets. These capabilities enable scalable, customizable, and reproducible analyses of plant datasets across diverse taxa and study designs. This flexibility offers unprecedented support for the discovery and description of the genetic and ecological factors that contribute to plant resilience and adaptation to environmental pressures.

Once a user has collected plants of interest from CartograPlant's Browse Page, the associated data can be downloaded or used to perform various forms of analysis on CartograPlant (Figs. 1c and 4). An analysis is a specific computational workflow which has been applied to a set of input data. HPC-supported workflows, implemented with NextFlow *v25.04.6* (Di Tommaso et al. 2017), are available within the Analysis Panel for the collected plants and/or studies. Registered users can create multiple workspaces to store data associated with various projects. A CartograPlant workspace consists of a directory holding raw data associated with a collection of individuals and, following an analysis, the generated outputs. Additional raw data and other input files necessary to run an analytic workflow of interest can be uploaded directly to a workspace from a user's local machine. Each workspace can be associated with one or more analyses. Both analyses and workspaces (and their input, intermediate, and resultant files) are private to users and can be saved for future use.

Currently supported analysis options are described in the following sections and include selection and correlation analysis of traits, marker overlap analysis, marker filtering, population structure inference, environmental layer data selection, GWAS, and GEA (Table 2; Fig. 4). To meet the needs of bioinformaticians, ecologists, land managers, and breeders, CartograPlant is continually updated with new analytic workflows as data, technologies, and methods evolve. Additionally, for analyses related to variant filtering, population structure, and marker association analyses (see Sections “Leveraging Genotypes Across Studies”, “Population Structure Inference”, and “Genotype-environment and Genotype-trait association” below) users can close the browser window while jobs run. Users can track the status of their current and past jobs across all workspaces using the Jobs link at the top of the page. The CartograPlant documentation contains instructional videos as well as connections to relevant repositories for the NextFlow workflows (see Data Availability).

### Study-level filtering

Within the Analysis Panel's Select Studies tab, a list of study titles associated with collected plants is displayed. For each study, counts of individuals, genotype calls, and phenotype measures are displayed. Users can remove entire studies from their collection before proceeding with analysis. The Select Studies tab draws from CartograPlant's standardized plant sample names such that if data about an individual is present in multiple collected studies, these data can be integrated and compared.

#### Phenotypes

Because CartograPlant enforces the mapping of phenotype measures to existing ontologies, trait data from multiple sources can be analyzed together. To facilitate trait-level exploration, the Analysis Panel's Filter Traits tab enables the selection of specific phenotypic measures from input data. A plot, generated with the R *v3.6.0* (R Core Team 2025) libraries *ggplot2* (Wickham 2011) and *ggbiplot* (Vu et al. 2024), is displayed including a two-dimensional trait Principal Component Analysis (PCA; Menozzi et al. 1978; Patterson et al. 2006) and an overlaid biplot with loading values for each of the selected traits. Users can view the displayed plot to determine which, if any, of their selected traits are correlated. Final user trait selection is saved for future use.

#### Leveraging genotypes across studies

Identifying shared genetic markers across studies is essential to synthesizing information across studies, expanding the scope of eco-evolutionary insight, and uncovering broad patterns of genetic variation across space and time. Because CartograPlant automatically remaps submitted markers to all available conspecific reference assemblies, including newly released assemblies as they are added, it is possible to merge all (or any subset of) marker data associated with a species into a single data structure. When studies represent markers mapped to disparate conspecific assemblies, CartograPlant's Select Genotypes tab allows users to leverage *bcftools* (Danecek et al. 2021) to merge them into a combined VCF mapped to a species' most recent reference assembly. This merged file is added to the current workspace and can be used in downstream analysis. In the future, users will be able to visualize marker remapping statistics across *all* available assemblies before choosing a reference for analysis (remapping implementation described in Supplementary File 1).

Across studies, various approaches for the identification of variant loci in a species can sometimes detect the same marker or utilize the same genotype panel. Within the Analysis Panel's Select Genotypes tab, statistics are displayed describing the intersection of marker sets among the collected studies. Two studies share a marker if (i) they both report a marker with the same reference assembly and locus or if (ii) they report markers on different assemblies that remap to the same locus on the most recent conspecific assembly. The accession of each collected study with marker data is shown along with the reference assembly (or reference assemblies) to which they were originally mapped. An UpSet plot illustrating overlaps among study marker sets is shown in Fig. 4.

The accurate detection of signals within genetic data that underlie plant adaptation and resilience depends on well-curated genotype datasets that minimize noise and bias. Rigorous filtering is essential to ensure that downstream GWAS and GEA analyses reflect true biological patterns rather than confounding effects such as population genetic structure (Rellstab et al. 2015; Sul et al. 2018). In the Analysis Panel's Filter Markers and Genotypes tab, genotype data quality indicators including percentage of missing data (both per marker and per individual) and minor allele frequency per marker can be displayed. Users can set thresholds for these statistics to filter markers at both the marker and individual level. The objective of SNP filtering is to remove markers, individuals, and genotype calls likely containing errors or insufficient data due to bioinformatic or wet lab processes (including errant sample labeling). Modular logical operators allow for any combination of these filters to be applied. The Filter Markers and Genotypes tab provides interactive histograms that allow users to select the best threshold values for the SNP quality filtering parameters.

Standard filtering processes include enforcement of a minimum genotype quality score to remove genotypes with poor instrumental confidence, a minimum allele count for each marker (i.e., half of the targeted coverage depth) to ensure well-supported genotype calls, a minimum percentage of missing genotypes per individual to remove individuals that were poorly genotyped, a threshold for missing genotype calls per marker across samples to retain only informative markers, and a minor allele frequency threshold to exclude alleles with insufficient statistical power (Pavan et al. 2020). In the Filter Markers and Genotypes tab, CartograPlant provides a GUI for constructing customizable modular *bcftools view* commands with logical operators to filter a VCF. When a user selects a statistic to begin defining a filter, a histogram of that statistic's distribution is generated to guide threshold choice. If the input VCF already contains pre-computed filter statistics in its header or INFO fields, those values can also be used directly for filtering. The underlying marker data distributions are retrieved with *bcftools query*, and the plots are produced with D3.js (Bostock et al. 2011).

#### Population structure inference

Inferring population genetic structure is a critical step in understanding how demographic history and genetic differentiation shape adaptive potential and resilience across landscapes. Population genetic structure refers to the presence of subgroups within a population exhibiting differences in allele frequencies shaped by factors including demographic history, isolation by distance, genetic drift, and kinship (Wright 1931, 1943, 1951). The results of many analyses of population-level data, including GWAS, can be skewed or biased if this structure is not considered (Pavan et al. 2020). To allow the inference of population structure from genetic datasets, the Analysis Panel's Assess Population Structure tab allows for the use of fastSTRUCTURE (Raj et al. 2014). STRUCTURE-like analyses are Bayesian approaches that calculate a posterior probability to estimate genetic structure (Pritchard et al. 2000; Falush et al. 2003, 2007; Hubisz et al. 2009). Prior to running fastSTRUCTURE, the genotypes are pruned based on linkage disequilibrium using PLINK (Purcell et al. 2007; Slifer 2018). Inferences from STRUCTURE-like approaches are highly dependent on the chosen number of subpopulations, *k* (Pritchard et al. 2000). CartograPlant allows users to estimate the degree of population structure across their selected accessions across a range of *k-*values. For each *k*, the workflow computes a *Q-*matrix and a set of marginal likelihoods and chooses a maximizing *k*. A global ancestry barplot, in which each individual is represented by a stacked bar reflecting the ancestry proportions in the best fastSTRUCTURE *Q*-matrix, is generated (Wickham 2011).

#### Environmental layers for georeferenced populations

Environmental variation is a major driver of plant distribution, ecology, evolution, and resilience. To enable analyses of genotypic variation within an environmental context, CartograPlant integrates a curated library of environmental layers with relevance to plant ecology, adaptation, and conservation. Environmental data from the location of each selected individual can be retrieved for analysis from CartograPlant's stored environmental layers using the Analysis Panel's Select Environmental Metrics tab. If environmental data is added to an Analysis that accompanies genotypic data, a user can perform GEA.

CartograPlant currently provides access to 964 North American and global environmental layers. These environmental layers fall into four broad categories: (i) biological, environmental, and climate data such as Normalized Difference Vegetation Indices for phenology estimation, delineations of areas of global human influence or biotic damage, aridity indices, and climate data from widely used sources such as ClimateWNA and WorldClim (Wang et al. 2016; Fick and Hijmans 2017); (ii) forest and vegetation data such as tree cover, canopy height, forest fragmentation indices, national forest boundaries, land use, and seed zones for the Eastern United States; (iii) biodiversity and species data including species ranges, biodiversity hotspots, protected areas, ecoregions, National Ecological Observatory Network (NEON) ecoclimatic domains, and tree species population data; and (iv) agriculturally cultivated and protected areas as well as soil data (Table 3). To add environmental data to an analysis, users can select specific layers and load these into a workspace (Fig. 4). CartograPlant uses the R package *psych (*Revelle 2025*)* to generate a trellis plot containing several statistics for visualizing relationships between selected environmental variables. This allows users to visually assess relationships among environmental variables and identify sets of variables that are relatively uncorrelated, which can be especially useful when preparing data for downstream analyses such as GEA modeling.

**Table 3.** CartograPlant hosts 964 environmental layers which can be classified into 4 broad categories.

Layer group	Extent	Description	Resolution	Source	Reference
**Biological, environmental, and climate data**					
**Biotic damage**	United States, Canada	Presence and damage caused by Emerald Ash Borer, Forest Tent Caterpillar, Gypsy Moth, Hemlock Woolly Adelgid, Winter Moth; total defoliation and mortality	variable	USFS	USDA n.d.-a
				UVM	Duncan et al. 2018
				EABIN	Emerald Ash Borer Information Network
**Climatic variables**	Global, North America	Temperature, precipitation, aridity, seasonality (temperature, precipitation, aridity), and soil types, temperature and permafrost probablity	1 km	Worldclim	Fick and Hijmans 2017
				ClimateNA	Wang et al. 2016
				FAO	.
				PANGAEA	Obu et al. 2018
** NDVI**	Global (FAO), conterminous United States (MODIS)	Vegetation health index (Normalized Difference Vegetation Index) derived from satellite imagry, and phenology metrics	variable (FAO), 250 m (MODIS)	NOAA-AVHRR MODIS USGS	Anyamba and Tucker 2005 Benedict et al. 2022 Benedict and Rigge n.d.
**Terrestrial Ecoregions**	Global	Global classification of terrestrial ecoregions	coarse	WWF	Olson et al. 2001
**NEON field sites**	United States (discrete sites)	Locations and monitoring data from the National Ecological Observatory Network	variable	NSF	National Ecological Observatory Network n.d.
**Low impact areas**	Global	Landscapes with low current human density and impacts not primarily managed for human needs	1 km		Jacobson et al. 2019
**Forest and vegetation data**					
**Forest fragmentation**	North America	Forest fragmentation assessment based on an area's forest cover and adjacent pixel forest cover	1 km	USGS	Riitters et al. 2000
**Land cover**	Global	Global land cover classification for vegetation and land use	30m	GLAD	Potapov et al. 2022
**National forests**	United States	Boundaries of U. S. national forests	variable	USDA	USDA n.d.-b
**Canopy height**	Global	Canopy height data	30 m 1 km	NASA	National Aeronautics and Space Administration n.d.; Potapov et al. 2022
				GLAD	Potapov et al. 2022
**Seed Zones**	Eastern United States	Delineation of seed zones of the Eastern United States	regional	USFS	Pike et al. 2020
**Intact forest landscape**	Global	Extent of the intact forest landscapes for years 2000, 2013, 2016, and 2020	scale 1:1,000,000	IFL	Potapov et al. 2017
**FIA land cover**	United States	County-level statistics on forest land cover, timber volume, biomass, carbon stock, and forest growth and removals, along with sampling errors	county	USDA	USDA n.d.-c
**Biodiversity and species data**					
**Biodiversity hotspots**	Global	Areas of high biodiversity and conservation priority	30 m	WRI	Global Forest Watch, and Conservation International n.d.
**Species range maps**	Global	Geographic distribution of plant species	NA	EUFORGEN	EUFORGEN n.d.
				USDA	Thompson et al. n.d.
				BIEN	Maitner et al. 2018
**Density population**	United States	Population density of various plant species within the United States	30 m	USDA	USDA n.d.-d
**PET and aridity**	Global	Global aridity index and evapotranspiration	30 arc-second		Zomer and Trabucco 2019; Zomer et al. 2022
**Agriculturally cultivated areas, protected areas, and soil data**					
**Protected and cultivated areas**	Global	National parks and equivalents which hold economic and scientific importance or which will be preserved in their natural state	variable	IUCN, UNEP-WCMC	UNEP-WCMC n.d.
**General soil types**	United States	Geographic distribution of soil types	1:12,000–1:63,360	USDA	USDA n.d.-d

Layer groups, which are sets of similar layers, are given. The extent, description, resolution, and source of each layer group is described.

Abbreviations: USGS = United States Geological Survey; USFS = United States Forest Service; EABIN = Emerald Ash Borer Information Network; FEMC = Forest Ecosystem Monitoring Cooperative; USDA = United States Department of Agriculture; FAO = Food and Agriculture Organization of the United Nations; EUFORGEN = European Forest Genetic Resources Programme; BIEN = Botanical Information and Ecology Network; GLAD = Global Analysis and Discovery; IFL = Intact Forest Landscapes; NSF = National Science Foundation; WWF = World Wildlife Foundation; NASA = National Aeronautics and Space Administration; WRI = World Resources Institute; IUCN = International Union for Conservation of Nature; UNEP-WCMC = United Nations Environment Programme World Conservation Monitoring Centre, UVM = University of Vermont.

#### Genotype-environment and genotype-trait association analysis

Marker association studies provide a powerful framework for the identification of the genetic variation underlying variability in phenotypic traits as well as adaptive responses to the environment, especially within analytical workflows that support the integration of diverse data types and user configurations. In genotype-phenotype analyses, genetic variation at individual loci or windows is associated with trait measures using models in which genotypes act as predictor variables and phenotypes as response variables. Within the Analysis Panel's Conduct Analysis tab, CartograPlant offers GUI-mediated access to two models from GEMMA (Genome-wide Efficient Mixed Model Association; Zhou and Stephens 2012, 2014): a basic linear model (LM) and a univariate linear mixed model (LMM). The LM tests associations between SNPs and traits without accounting for confounding effects, while the univariate LMM extends this framework by incorporating both fixed effects and random effects that model sample relatedness and population structure. The LMM can also estimate the proportion of variance explained by SNP heritability in a phenotype. By supporting both models, CartograPlant enables users to perform GWAS that range from simple association tests to more robust analyses that account for population structure and relatedness.

To complement genotype-phenotype analyses, Cartograplant implements genotype-environment analysis using LFMM2 from the R package LEA (Caye et al. 2019). LFMM2 implements a latent factor mixed model (LFMM) to perform GEA analysis. This LFMM implementation allows for the control of confounding effects, such as population structure, in the dataset. Specifically, LFMM2 uses a least-squares estimation approach for confounder estimation that provides a unique framework for several categories of genomic data. Upon completion, *P*-value results of a GWAS or GEA analyses are displayed within a Manhattan plot generated with the R package *ggplot2*. This plot groups markers by chromosome and allows for the visualization of markers with high association signals.

Statistical methods which involve the testing of many hypotheses, including GWAS and GEA, often require a multiple testing correction of the resultant *P*-values. As the number of association tests performed grows larger, so too does the probability that one or more results are representative of a Type 1 error (Moskvina and Schmidt 2008; Rellstab et al. 2015). To account for this, *P*-values generated under multiple hypothesis testing can be adjusted using a variety of techniques.

## Data-driven approaches to plant adaptation and conservation

### Enabling GWAS and GEA across studies though meta-analysis in model systems

*Populus trichocarpa*, a widely recognized model for bioenergy research, exhibits extensive latitudinal variation across western North America with substantial evidence for local adaptation to the climatic conditions of its native habitat (Evans et al. 2014; McKown et al. 2014a; Zhang et al. 2019). As a result, *P. trichocarpa* has emerged as a model species for genetics and plant biology research (Tuskan et al. 2006; Taylor et al. 2019), and has been the focus of numerous GWAS and GEA studies. These investigations have explored natural variation in phenology, wood properties, and traits related to lignin biosynthesis. Since the publication of the first reference genome in 2006, four major reference assemblies, along with several intermediate versions and updated annotations, have been released through the Phytozome data portal (Goodstein et al. 2012), each supporting new studies (Tuskan et al. 2006; Sreedasyam et al. 2023). Across these investigations, researchers have applied a range of genotyping and sequencing approaches, including genotyping assays (Geraldes et al. 2013, 2014; McKown et al. 2014b, 2014c), reduced representation methods such as exome capture (Zhou et al. 2014; Guerra et al. 2019), and whole genome resequencing (Slavov et al. 2012).

Many of these studies have relied upon a single, well-characterized common garden experiment. Despite this shared foundation, differences in marker systems, reference genome versions, and annotation frameworks make it challenging to integrate results across studies. Integrating these datasets allows users to identify consistent genotype-phenotype-environment relationships across studies, even when experimental designs and genomic resolutions differ. As a result, the cumulative impact of nearly two decades of research has not been fully realized. CartograPlant seeks to address this by enabling the biocuration of a subset of over 400 studies, systematically mapping variants across reference versions, standardizing trait metadata, and linking findings with spatial and temporal environmental data layers. This integrated framework enhances the reuse of legacy data and supports discovery of trait and environment associations.

### Mobile applications to connect traits, environment, and genotypes for plant resilience

Citizen science has emerged as a powerful resource for plant biologists, and enables the collection of large-scale ecological and trait data that would otherwise be logistically and financially infeasible. The growing participation of the public underscores the need for platforms to translate such observations into actionable resources, particularly for non-model plant species. This is especially true for species of conservation concern. For example, all *Fraxinus* (ash tree) species native to North America are under exceptional threat from the invasive Emerald Ash Borer (EAB, *Agrilus planipennis*). The conservation and restoration of ash species is a coordinated effort involving federal and state agencies, non-profit organizations, academic partners, and citizen scientists. These efforts focus on identifying lingering individuals that may exhibit resistance, understanding stand level dynamics, and documenting ecological pressures. This work integrates data from federal, state, and county level EAB surveys, citizen science contributions, and landowner observations, alongside the collection of plant material for seed banking, *ex situ* preservation, and the establishment of long-term monitoring plots.

CartograPlant provides a framework for these efforts by serving as the integration hub between mobile applications and genomic resources. Currently, TreeSnap (Crocker et al. 2020), a mobile citizen science application designed for species of forest health concern, enables custom surveys that capture both individual and stand-level data. In the case of ash, TreeSnap hosts survey modules tailored for both general users and experts who can provide more detailed information on EAB progression. In addition, CartograPlant has begun to incorporate data from MaMA (Monitoring and Managing Ash; https://www.monitoringash.org/), an independent assessment program that collects both qualitative and quantitative data from regional monitoring plots. Traits collected through TreeSnap and MaMA, can be connected to genotype data on a subset of the phenotyped accessions through TPPS.

### Eco-evolutionary dynamics in rapidly changing climates

Climate change is rapidly reshaping species distributions, altering community dynamics and species interactions, and exerting strong selection pressure on organisms that can cascade to ecosystem-level effects (Bailey et al. 2009). To understand and ameliorate these impacts, researchers increasingly emphasize the integration of ecological and evolutionary perspectives (Bolnick et al. 2011; Müller 2017; Brady et al. 2019; Fronhofer et al. 2023; Fouqueau and Polechová 2024).

For instance, the Arctic, which is warming four times faster than global norms (ACIA 2004; Hobbie et al. 2017), offers a uniquely tractable system to study evolutionary responses across multiple levels of biological organization under this eco-evolutionary framework. With relatively low species diversity and a strong coupling between biotic and abiotic processes, even subtle shifts could result in cascading effects on Arctic ecosystem structure and function (Wookey et al. 2009). Research on Arctic stream-riparian systems—particularly those dominated by keystone willow (*Salix*) species—are shedding light on how adaptive evolution influences ecosystem function. These stream-riparian systems are sensitive to ongoing climate change (Myers-Smith et al. 2011; Frost and Epstein 2014; Criado et al. 2020; Anderson et al. 2024), with potential cascading effects across trophic levels (Hollister et al. 2015; Zhou et al. 2020; Mekonnen et al. 2021). To support such investigations, CartograPlant aids in linking field observations, *in situ* organismal traits, common gardens, genetic sampling, and pertinent environmental data layers. For example, layers associated with permafrost, ground temperature, and hydrology have been incorporated to support this integration. Alongside, standardized, long-term environmental and ecological data from the NEON, these layers together provide the foundation for assessing ecological and evolutionary processes in *Salix* and other Arctic species, including rates of hybridization, environmental clines in trait and genetic diversity, as well as shifts in ploidy.

## Summary and future directions

CartograPlant streamlines the integration and analysis of diverse biological data by adhering to FAIR principles and standardized ontologies. It offers flexible, quality-controlled workflows for data filtering, analysis and visualization, facilitating reproducible insights into plant resilience, adaptation, and demography. Designed to evolve alongside emerging technologies and data sources, CartograPlant supports future integration of structural variation, pangenomes, high-throughput phenotyping data, as well as connections with primary repositories, ensuring its continued relevance in the rapidly advancing fields of ecology and evolution. For example, CartograPlant is actively developing two-way data integration with the European Variation Archive (Cezard et al. 2022), enabling automatic synchronization of variant datasets across platforms. We are also working to expand available climate layers to include sources such as CHELSA-climate.org and are actively working to incorporate gene and functional annotation into data and Analysis output to provide users with biological context for interpreting their results. This interoperability will further streamline data discovery, ensure that dataset identifiers are mappable between systems, and reduce duplication of biocuration effort. Because CartograPlant has the ability to ingest environmental and phenotypic data as well as detailed study metadata in addition to variant data, this collaboration has the potential to increase the amount of information available about a given variant dataset.

By integrating genotypic, phenotypic, and environmental data alongside a user-friendly interface, CartograPlant advances our understanding of biodiversity, ecological responses, and the traits that shape plant evolution in the face of environmental change and biotic stressors.

## Supplementary Material

iyag060_Supplementary_Data

## Data Availability

CartograPlant is available online at https://cartograplant.org. All data used in this study are publicly available through the TreeGenes database (https://treegenesdb.org). A tutorial for the submission of data through TPPS is available at https://gitlab.com/TreeGenes/tpps/. Documentation, tutorials, descriptions of expected run times, and additional information for CartograPlant can be found at https://cartograplant-tpps.readthedocs.io/en/latest/. These tutorials use data from studies in CartograPlant to illustrate the inputs, analytic workflows, and outputs. Code for NextFlow pipelines are available on GitLab (https://gitlab.com/TreeGenes) in the following repositories: gwas-pipeline, population-structure-pipeline, variant-filtering-pipeline. These repositories include documentation of the workflows' implementation and usage, and enable export to other computing environments. The demonstration datasets in Fig. 4 can be accessed from CartograPlant via accessions: TGDR 1892 (Geraldes et al. 2013) and TGDR 2269 (McKown et al. 2014c). Supplemental material available at GENETICS online.
