# Supplementary material for: CartograPlant: bridging genomic, phenotypic, and environmental data to advance plant resilience and eco-evolutionary insight
Source: Genetics. 2026 Mar 6;232(4):iyag060. doi: 10.1093/genetics/iyag060 (PMC13050189; doi:10.1093/genetics/iyag060)
Supplement: iyag060_Supplementary_Data [file iyag060_supplementary_data.docx]

Supplemental File S1

**CartograPlant: Bridging genomic, phenotypic, and environmental data to advance plant resilience and eco-evolutionary insight**

Brandon M. Lind^1,2*†^, Irene Cobo-Simón^1,3†^, Meghan Myles^1†^, Gabe Barrett^1^,
Emily Grau^1^, Risharde Ramnath^1^, Vlad Savitsky^1^, Jill L. Wegrzyn^1,2*^

^1^Department of Ecology and Evolutionary Biology, University of Connecticut, Storrs, CT 06269, USA

^2^Institute for Systems Genomics, University of Connecticut, Storrs, CT 06269, USA

^3^Departamento de Ecología y Genética Forestal, Instituto de Ciencias Forestales (ICIFOR), Instituto Nacional de Investigación y Tecnología Agraria y Alimentaria - Consejo Superior de Investigaciones Científicas (ICIFOR-INIA, CSIC), Madrid, Spain

30 January 2026

**Running title**: *Eco-evolutionary insight with CartograPlant*

**Keywords**: data integration, data interoperability, meta-analysis, biodiversity informatics, plant adaptation

^†^These authors contributed equally

***Corresponding Authors**

Brandon M. Lind

ORCID: 0000-0002-8560-5417

Email: [lind.brandon.m@gmail.com](mailto:lind.brandon.m@gmail.com)

Jill L. Wegrzyn

ORCID: 0000-0001-5923-0888

Email: [jill.wegrzyn@uconn.edu](mailto:jill.wegrzyn@uconn.edu)

## Supplemental Text - Automated marker remapping

CartograPlant performs automated remapping of all marker data to all conspecific reference assemblies, including new assemblies as they are generated. It uses a custom Python (Rossum 1995) v3.6.8 script which wraps a number of common bioinformatics tools to perform flank-based marker remapping. This script takes, as input, genotype data in either VCF or tabular format as well as a FASTA assembly to remap them to. It generates a VCF which contains genotype data as mapped to a different assembly. The following text will describe the software and methods used to complete this process.

The custom Python script initially computes indices for the target reference FASTA, using samtools faidx (Danecek et al., 2021) and bwa index (Li 2013). Then, the script checks whether it has been given marker flank sequence data. If not, for each SNP, it uses samtools faidx (Danecek et al., 2021) to obtain an 81-bp sequence from the reference genome to which the markers were initially mapped. This sequence represents the 40 nucleotides before the SNP, the SNP itself, and the 40 nucleotides after the SNP. If marker flank sequence data was given as an input, no new flanks are obtained.

The script then preprocesses SNP flank sequences by changing the nucleotide at the position of the variant to an N. Next, it creates a FASTA containing all of the SNP flank sequences. It uses a default bwa mem (Li 2013) command to align the flank sequence FASTA to the new assembly version FASTA. It then uses samtools view to parse the output binary alignment map (BAM) and samtools sort to sort it. It indexes the BAM with samtools index (Danecek et al., 2021) and converts it to a Browser Extensible Data (BED) file with bedtools bamtobed (Quinlan and Hall 2010). It obtains remapped marker positions from the BED file and stores them within a pandas (The pandas development team 2019) dataframe. It then uses samtools faidx (Danecek et al., 2021) to determine the new reference allele for each marker from the assembly to which it is being remapped and adds it to the dataframe. For each SNP, it obtains a list of all called alleles in the input dataset, subtracts from this set the new reference allele, and adds this list to the dataframe as the possible alternate alleles. Finally, the genotype calls themselves are added to this dataframe, and numeric SNP calls are updated to reflect each SNP’s new reference and alternate alleles.

The Python script then uses the generated dataframe to create a VCF. In this way, flank-based marker remapping can be completed for the integration of genotypic datasets which are mapped to different reference assemblies.

## References

Danecek, P., J. K. Bonfield, J. Liddle, J. Marshall, V. Ohan, M. O. Pollard, A. Whitwham, T. Keane, S. A. McCarthy, R. M. Davies, and H. Li. 2021. Twelve years of SAMtools and BCFtools. GigaScience 10:giab008.

Li, H. 2013. Aligning sequence reads, clone sequences and assembly contigs with BWA-MEM.

Quinlan, A. R., and I. M. Hall. 2010. BEDTools: a flexible suite of utilities for comparing genomic features. Bioinformatics 26:841–842.

Rossum, G. 1995. Python reference manual. CWI (Centre for Mathematics and Computer Science), NLD.

The pandas development team. 2019. pandas-dev/pandas: Pandas. Zenodo. https://doi.org/10.5281/zenodo.3509134
